# First case of somatic mosaicism in TRAPS caused by a novel 24 nucleotides deletion in the TNFRSF1A gene

**DOI:** 10.1186/1546-0096-13-S1-O60

**Published:** 2015-09-28

**Authors:** D Rowczenio, E Omoyinmi, H Trojer, T Lane, P Brogan, P Hawkins, H Lachmann

**Affiliations:** 1 National Amyloidosis Centre, University College London, London, UK; 2 Institute of Child Health, UCL, London, UK

## Introduction

TNF receptor associated periodic syndrome (TRAPS) is an autosomal dominant disease caused by gain-of-function mutations in the TNF superfamily receptor 1A (*TNFRSF1A*) gene encoding 55 kDa TNF receptor type I (TNFR1). TRAPS is characterized by episodes of fever accompanied by severe abdominal pain, arthralgia, myalgia, rash, chest pain, enlarged glands and red, swollen eyes. Duration of the attacks can range from few days to several weeks, with the onset from early childhood to adulthood.

## Objective

To identify the cause of fever in a 41 year old man who suffered with symptoms of severe abdominal pain, headache, arthralgia, myalgia, night sweats, generalised erythema and unilateral non-painful cervical lymphadenopathy which started from early adolescence, but became more severe after he recently returned from working overseas. He had 10 and 12 attacks per year each lasting almost exactly two weeks.

## Methods

The patient underwent screening of the four genes: *MEFV* (the gene associated with FMF); *TNFRSF1A* (the gene associated with TRAPS) *NLRP3* (the gene associated with CAPS) and *MVK* (the gene associated with MKD).

DNA was extracted from whole blood, saliva, buccal epithelial cells, hair root and from isolated monocytes, T and B lymphocytes and neutrophils.

## Result

A novel in-frame deletion of 24 nucleotides (c.255_278del) in exon 3 of the *TNFRSF1A* gene was identified by PCR and Sanger sequencing in DNA extracted from whole blood, but the size of mutated nucleotide peaks on the chromatogram were notably smaller than wild-type, raising the possibility of mosaicism. The latter was confirmed by targeted sequencing, which established the frequency of the mutant allele in the DNA isolated from whole blood as 7.36% and the virtual absence of variant sequence in purified epithelial cells (buccal swab), but substantial representation of the mutation in T lymphocytes and neutrophils. Analysis of parental DNA showed only wild-type *TNFRSF1A* gene sequence, corroborating that the deletion identified in our patient had occurred *de novo*.

## Conclusion

We report first mosaic patient with TRAPS caused by a novel *TNFRSF1A* deletion of 24 nucleotides found in 7.36% of cells. The patient responded extremely well to treatment with anakinra, including a complete remission of symptoms and normalization in SAA and CRP.

The deletion of highly conserved residues, including the F60 amino acid, which is crucial for proper protein folding, is likely to introduce profound changes to the function and three dimensional shape of the TNFR1 potentially impairing its folding and binding with TNF.

